# The Association between Earlobe Crease (Frank's Sign) and Abnormal Ankle-Brachial Index Determination Is Related to Age: A Population-Based Study

**DOI:** 10.1155/2018/4735731

**Published:** 2018-09-02

**Authors:** Oscar H. Del Brutto, Robertino M. Mera, Aldo F. Costa, Mauricio Zambrano, Mark J. Sedler

**Affiliations:** ^1^School of Medicine, Universidad Espíritu Santo-Ecuador, Guayaquil, Ecuador; ^2^Vanderbilt University Medical Center, Nashville, TN, USA; ^3^Community Center, The Atahualpa Project, Atahualpa, Ecuador; ^4^School of Medicine, Stony Brook University, New York, NY, USA

## Abstract

**Background:**

Information on the association between earlobe crease (ELC) and peripheral artery disease is limited. We assessed this association in community-dwelling older adults.

**Study Design:**

A total of 294 Atahualpa residents aged ≥60 years were enrolled. ELC were visually identified by two raters. The ankle-brachial index (ABI), used as a surrogate of peripheral artery disease, was categorized using American Heart Association criteria. Using logistic regression and probability models, adjusted for demographics and cardiovascular risk factors, we assessed the relationship between ELC and abnormal ABI determinations, as well as the influence of age on this association.

**Results:**

ELC was identified in 141 (48%) individuals, and abnormal ABI determination was carried out in 56 (19%). The association between ELC and abnormal ABI was nonsignificant in logistic regression and probability models with individuals stratified according to their median age.

**Conclusions:**

The association between ELC and abnormal ABI determinations is probably attenuated by the high prevalence of both conditions in older persons. ELC might not be useful for identifying candidates for ABI determination.

## 1. Introduction

Earlobe crease (ELC), also known as Frank's sign, is a wrinkle extending from the tragus to the outer border of the earlobe [1]. Studies have linked ELC with cardiovascular events [2, 3]. However, others believe that ELC may be an unrelated bystander [4]. ELC has also been associated with increased carotid artery intima-media thickness and arterial stiffness [5]. These findings have opened new venues for the use of ELC for identifying apparently healthy individuals at risk of developing vascular events.

A recent study showing that ELC may detect asymptomatic individuals with peripheral artery disease (PAD) is promising [6], since untreated PAD has been associated with foot ulcers, lower limb amputations, cardiovascular events, and all-cause mortality [7]. However, information on the predictive value of ELC for detection of subjects with PAD is limited and may not be representative of the population at large. Here, we aimed to assess the relationship between ELC and abnormal ABI determinations in community-dwelling older adults living in rural Ecuador (Atahualpa).

## 2. Material and Methods

Atahualpa represents an optimal setting for the practice of epidemiological studies. As previously described, villagers are homogeneous regarding race/ethnicity, lifestyle, socioeconomic status, and diet [8]. The IRB of Hospital-Clínica Kennedy, Guayaquil, Ecuador (FWA 00006867), approved the protocol and the informed consent. All Atahualpa residents aged ≥60 years were invited to participate for evaluating the association between the presence and characteristics of ELC and results of ABI determinations, after adjusting for relevant confounders.

**Figure 1:**
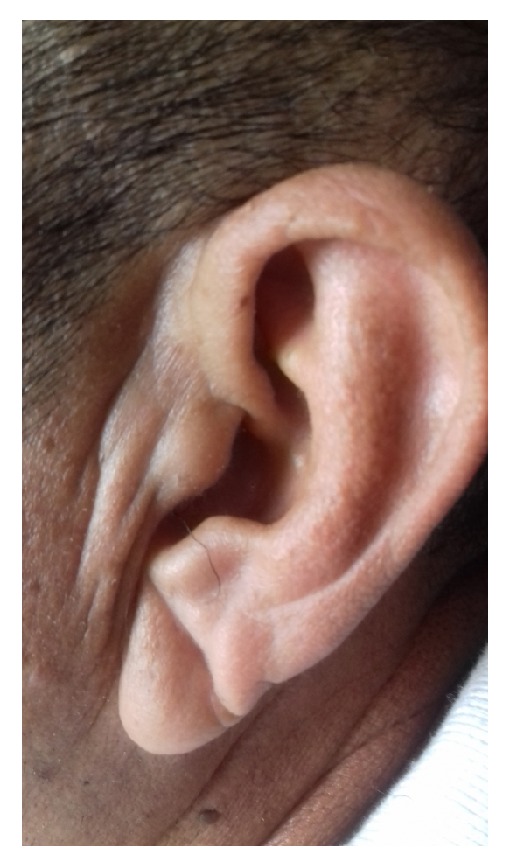
Diagonal earlobe crease in an Atahualpa resident.

Both earlobes were examined with the subject in the sitting position. According to Rodriguez-Lopez et al. [9], an ELC was considered to be present when the individual has a wrinkle (of different shapes and length) extending from the tragus to the outer border of the earlobe (Figure 1). Subjects with creases related to earrings and those who have physical damage distorting earlobe anatomy were excluded. Two investigators, blinded to each other's assessments and to clinical data assessed the earlobes. Interrater agreement was adequate for ELC presence (kappa=0.95), and discrepancies were resolved by consensus.

ABI determinations followed the recommendations of the American Heart Association [7]. A manual sphygmomanometer and a portable vascular Doppler with an 8 MHz probe were used for blood pressure (BP) measurements. The ABI was calculated by dividing the highest of the posterior tibial or dorsalis pedis BP in each leg by the higher arm systolic BP (right or left). The lowest ABI between the two ankles was used for classifying purposes. ABI determinations ≤0.9 and ≥1.4 were considered abnormal, the former reflecting PAD and the latter calcinosis.

Demographics and cardiovascular risk factors, selected as confounding variables, were assessed through interviews and procedures previously described in the Atahualpa Project. These included age, sex, smoking status, physical activity, diet, the body mass index, BP, fasting glucose, and total cholesterol blood levels [10].

Data analyses are carried out by using STATA version 14 (College Station, TX, USA). In univariate analyses, continuous variables were compared by linear models and categorical variables by *x*^2^ or Fisher exact test as appropriate. Using logistic regression models, we evaluated whether ELC was associated with an abnormal ABI (dependent variable), after adjusting for demographics and cardiovascular risk factors. We anticipated a potential effect modification of age in the association between ELC and abnormal ABI determinations and fitted multivariate probability models with participants stratified according to the median age of the population.

## 3. Results

Of 437 Atahualpa residents aged ≥60 years identified during door-to-door surveys, 294 (67%) underwent ABI determination and earlobe examinations. Of the 143 nonincluded individuals, earlobe deformities did not allow characterization of ELC in 14, and the ABI could not be obtained in 72 cases because of refusal to consent (n=57), leg amputations (n=10), and uncontrolled tremor in the legs (n=5). In addition, 57 subjects had died or emigrated between enrollment and the invitation.

Table 1 shows the characteristics of participants across categories of ABI. As noted, individuals with abnormal ABI determinations were older, had worse physical activity, and had more often BP levels ≥140/90 mmHg than those with normal ABIs. Regarding ELC, univariate analysis showed a nonsignificant trend for being more frequent among individuals with abnormal ABI determinations (p=0.068).

**Figure 2:**
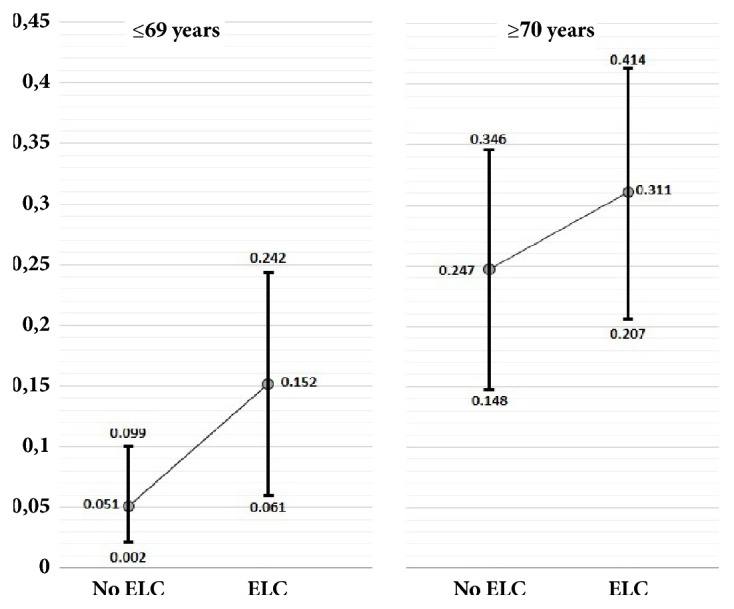
Probability margins of abnormal ankle index (ABI) determination according to earlobe crease (ELC) presence, with participants stratified by the median age of the population (69 years). In both age groups, the probability of abnormal ABI is higher among individuals with an ELC, but differences are not significant due to overlapping 95% confidence intervals.

A logistic regression model, adjusted for demographics and cardiovascular risk factors, revealed no significant association between ELC and abnormal ABI determinations (OR: 1.71; 95% CI: 0.89 – 3.26; p=0.104). In this model, the only significant covariate was age (p<0.001). Probability margins of abnormal ABI determinations, with individuals stratified according to their median age (69 years), showed that probabilities of abnormal ABI determinations nonsignificantly increased among older individuals with ELC (Figure 2).

## 4. Discussion

Results of this study suggest that the association between ELC and abnormal ABI determinations could be attributed to age. These findings might be explained by the high prevalence of both variables in older adults, confirming previous hypotheses that ELC and atherosclerosis could be related to the effects of aging [4, 11].

In a study aimed at correlating ELC with ABI determination [6], 136 of 253 (54%) individuals free of symptomatic atherosclerosis had ELC, and ABI determinations were significantly lower than in those without ELC. However, the authors used a different ABI cutoff value for defining PAD and results may not be comparable with our findings. Moreover, the influence of age in the relationship between ELC and abnormal ABI determinations was not specifically investigated. Yet, in another study, the authors found no significant association between the presence of an ELC and peripheral artery disease [12].

There is limited information about pathogenetic mechanisms explaining how ELC might correlate with atherosclerosis. In an autopsy-based report, it was suggested that* “one of the underlying pathogenetic mechanisms involved in the progression of atherosclerosis, possible related to collagen metabolism, may also occur in the skin”* [13]. It has also been suggested that the ELC might be genetically determined and related to the occurrence of atherosclerosis, which might explain its different prevalence according to race/ethnicity [2].

The present study has limitations. Results may not be generalized to other races/ethnic groups but are of value for Amerindians, a population where the association between ELC and atherosclerosis has not been previously investigated. In addition, we cannot rule out the presence of some hidden confounders (such as asymptomatic coronary artery disease), which can modify the final results obtained in the multivariate model. Also, the possibility of a statistical type 2 error cannot be completely ruled out, because the statistical power of our sample was 57.5%. Major strengths include the population-based design with unbiased enrollment of participants and the models used to evaluate the influence of age on the association between ELC and abnormal ABI determinations.

## 5. Conclusions

This study suggests that the association between ELC and abnormal ABI determinations is attenuated by the high prevalence of both conditions in older persons. ELC might not be useful for identifying candidates for ABI determination.

## Figures and Tables

**Table 1:** Characteristics of Atahualpa residents aged ≥60 years across categories of ankle-brachial index determinations (univariate analyses).

	Total series (n=294)	Normal ABI (n=238)	Abnormal ABI (n=56)	*p* value
Age, years (mean ± SD)	70.9 ± 7.9	69.6 ± 6.9	76.5 ± 9.3	<0.001
Women, n (%)	171 (58.2)	138 (58)	33 (58.9)	0.897
Current smokers, n (%)	4 (1.4)	4 (1.7)	0	0.741
Body mass index ≥30 kg/m^2^, n (%)	64 (21.8)	53 (22.3)	11 (19.6)	0.668
Poor physical activity, n (%)	23 (7.8)	14 (5.9)	9 (16.1)	0.011
Poor diet, n (%)	13 (4.4)	8 (3.4)	5 (8.9)	0.079
Blood pressure ≥140/90 mmHg, n (%)	131 (44.6)	99 (41.6)	32 (57.1)	0.035
Fasting glucose ≥126 mg/dL, n (%)	96 (32.7)	78 (32.8)	18 (32.1)	0.928
Total cholesterol ≥240 mg/dL, n (%)	37 (12.6)	27 (11.3)	10 (17.9)	0.671
Presence of earlobe crease, n (%)	141 (48)	108 (45.4)	33 (58.9)	0.068

## Data Availability

The data is confidential according to the policy of the authors' institution.
